# Effect of Alanine Replacement of L17 and F19 on the Aggregation and Neurotoxicity of Arctic-Type Aβ_40_

**DOI:** 10.1371/journal.pone.0061874

**Published:** 2013-04-25

**Authors:** Yi-Ru Chen, Hsien-bin Huang, Chi-Jen Lo, Chih-Ching Wang, Li-Kang Ho, Hsin-Tzu Liu, Ming-Shi Shiao, Ta-Hsien Lin, Yi-Cheng Chen

**Affiliations:** 1 Structural Biology Program, National Yang-Ming University, Taipei, Taiwan, R.O.C; 2 Department and Institute of Pharmacology, National Yang-Ming University, Taipei, Taiwan, R.O.C; 3 Department of Life Science and Institute of Molecular Biology, National Chung Cheng University, Chia-Yi, Taiwan, R.O.C; 4 Department of Medical Research and Education, Taipei Veterans General Hospital, Taipei, Taiwan, R.O.C; 5 Institute of Biochemistry and Molecular Biology, National Yang-Ming University, Taipei, Taiwan, R.O.C; 6 Voiding Dysfunction Therapeutic Center in the Research Department, Buddhist Tzu Chi General Hospital, Hualien, Taiwan, R.O.C; 7 Department of Life Science, Chang Gung University, Kwei-Shan Tao-Yuan, Taiwan, R.O.C; 8 Department of Medicine, MacKay Medical College, New Taipei City, Taiwan, R.O.C; Cambridge Institute for Medical Research, United Kingdom

## Abstract

Alzheimer’s disease is the most common form of neurodegenerative disease. Beta-amyloid peptides (Aβ) are responsible for neuronal death both *in vitro* and *in vivo*. Previously, L17 and F19 residues were identified as playing key roles in the stabilization of the Aβ_40_ conformation and in the reduction of its neurotoxicity. In this study, the effects of L17A/F19A mutations on the neurotoxicity of Aβ genetic mutant Arctic-type Aβ_40_(E22G) were tested. The results showed that compared to Aβ_40_(E22G), Aβ_40_(L17A/F19A/E22G) reduced the rate of conformation conversion, aggregation, and cytotoxicity, suggesting that L17 and F19 are critical residues responsible for conformational changes which may trigger the neurotoxic cascade of Aβ. Aβ_40_(L17A/F19A/E22G) also had decreased damage due to reactive oxygen species. The results are consistent with the discordant helix hypothesis, and confirm that residues 17–25 are in the discordant helix region. Compared to Aβ_40_(L17A/F19A), reduction in aggregation of Aβ_40_(L17A/F19A/E22G) was less significantly decreased. This observation provides an explanation based on the discordant helix hypothesis that the mutation of E22 to G22 of Aβ_40_(E22G) alters the propensity of the discordant helix. Arctic-type Aβ_40_(E22G) aggregates more severely than wild-type Aβ_40_, with a consequential increase in toxicity.

## Introduction

Alzheimer’s disease (AD) is the most common neurodegenerative disease in the elderly population [1]–[3]. There are two forms of AD, late onset sporadic (SAD) and early onset familial (FAD) [4]. SAD is predominantly diagnosed in people over 65 years of age, and less frequent FAD often occurs in patients under the age of 65 years [5], [6]. FAD is clinically considered the most serious form of AD. Regardless of the form of AD, amyloid senile plaques (SPs) and neurofibrillary tangles (NFTs) are the two most important pathological hallmarks in the brains of AD patients [7]. In SPs, the main component is the β amyloid peptide (Aβ), that is either 40 (Aβ_40_) or 42 (Aβ_42_) amino acids long, containing hydrophobic amino acid sequences at its C-termini. This peptide is the proteolytic product of the amyloid precursor protein (APP), which is sequentially cleaved first by β-secretase, then by γ-secretase [8], [9].

Amyloid deposits of Aβ peptides for both SAD and FAD, including oligomers, protofibrils, and fibrils have been demonstrated to be toxic to neural cells, and are the main causative agents leading to AD [10], [11]. The neurotoxicity induced by Aβ aggregates has been associated with formation of reactive oxygen species (ROS) and reactive nitrogen species (RNS) [12], [13]. Furthermore, neurotoxicity is highly correlated with its structural and molecular states [14]. Fibrilligenesis of Aβ is usually accompanied by a conformational conversion from either α-helix or random coil to β-sheet/strands during the aggregation process [15], [16]. The conformation of Aβ is well correlated with sequence and toxicity. Using different Aβ fragments or truncated Aβ peptides, several sequence regions, including residues 17–20, 30–35, and 41–42, have been shown to play roles in conformation and toxicity [17]–[19].

For FAD, several hereditary mutations have been identified [20]. Flemish-type Aβ(A21G) [21], Arctic-type Aβ(E22G) [22], [23], Dutch-type Aβ(E22Q) [24], Italian-type Aβ(E22K) [25], and Iowa-type Aβ(D23N) [26] are the most well-known. Few studies have characterized the folding, aggregation, and toxicity of Arctic-type Aβ(E22G) [27]–[29]. Compared with wild-type Aβ_40_, Arctic-type Aβ(E22G) shows a higher propensity to form β-strand structures and exhibit rapid aggregation, with accompanying severe toxicity. By creating mutants that can inhibit conformational change and result in stable secondary structures, it may be possible to identify key residues that affect the processes of aggregation and conformational change.

To reduce toxicity induced by Aβ, it is necessary to prevent structural conversion and aggregation of Aβ [15], [16], [30]. Several studies have suggested that residues 16–23 constitute a region of the discordant helix [30]–[34], and any factors which stabilize the conformation of this region can prevent the aggregation and reduce toxicity of Aβ_[40]_ [32], [35]. Our previous studies found that 17–25 is the discordant helix region that can stabilize Aβ structure and inhibit aggregation [36]. This identification provides the basis for determining key residues that can increase helical propensity. Previously, we identified L17 and F19 as key amino acids in the stabilization of the Aβ_40_ conformation. By constructing an Aβ_40_(L17A/F19A) mutant, we further demonstrated that replacement of L17 and F19 with alanine can stabilize the Aβ conformation, reduce Aβ aggregation, and diminish Aβ-induced neurotoxicity [37]. Most Aβ peptides of FAD contain one point mutation of wild-type Aβ [21]–[26]. Some of them, such as Arctic-type Aβ, can cause more severe cell death than wild-type Aβ. Among these familial Aβ mutants, the Arctic-type Aβ has been shown to accelerate the development of clinical and pathological features indistinguishable from those of sporadic AD.

To further investigate the effects of L17A/F19A mutations on Aβ properties, we used the same strategy as a previous study [37] and constructed an Aβ_40_(L17A/F19A/E22G) mutant to characterize and compare the related properties with the genetic Arctic mutation of Aβ_40_(E22G), which has been shown to be the most toxic FAD Aβ mutant [23]. We wished to determine whether the L17A/F19A mutations can result in stable conformations and less neurotoxicity not only in wild-type Aβ, but also in FAD. *In vitro* studies were used to show that the L17/F19 mutants decreased Aβ aggregation and changed aggregation morphology. *In vivo* studies were used to verify neurotoxicity by using a cell viability assay. Our results show that compared to Aβ_40_(E22G), Aβ_40_(L17A/F19A/E22G) can reduce aggregation and neurotoxicity. These results demonstrate that replacement of L17 and F19 with alanine residues decreases aggregation and neurotoxicity of Aβ_40_(E22G), further suggesting that L17 and F19 are key residues in the stabilization of Aβ. Our study also verifies the identification of critical residues responsible for conformational changes which may trigger the neurotoxic cascade of Aβ_40_ and its genetic mutations.

## Materials and Methods

### Aβ Peptide Preparation

Production of recombinant Aβ peptides used the cloning protocol as previously described [38]. cDNAs of Aβ_40_ were a kind gift from Professor Paul Greengard. *Eschericha coli* BL21(DE3) (Sigma, St. Louis, USA) was used for expression. All Aβ peptides were purified on a reverse phase C_18_ HPLC column (Waters, Milford, Massachusetts, USA) with a linear gradient from 0% to 100% acetonitrile. The molecular weight of the purified Aβ peptides was verified by MALDI-TOF mass spectroscopy. They were freshly prepared in a 1 mM stock solution in 2,2,2- trifluoroethanol (Sigma, St. Louis, USA).

### Circular Dichroism (CD) Spectroscopy and Secondary Structure Analyses

Far-UV CD spectra were collected from 190–260 nm at 37°C using a synchrotron radiation circular dichroism (SRCD) spectropolarimeter at the 04B1 beam station of the national synchrotron radiation center in Taiwan. For SRCD measurements, a final peptide concentration of 60 µM in 20 mM phosphate buffer, pH 7.0, was used. All measurements were performed in CaF_2_ cells with a path length of 0.1 mm. Each SRCD spectrum was reported as the average sum of three separate analyses. Secondary structure analysis was performed in an online web server Dichroweb [39], [40] using the CDSSTR program.

### Thioflavin-T Peptide Aggregation Assay

The peptide stock solution was dried under N_2_ gas and resuspended in 20 mM phosphate buffer, pH 7.0, to a final concentration of 60 µM. Thioflavin T (Th-T) (Sigma, St. Louis, USA) dye (30 µM) was added to the freshly prepared peptide solution at a molar ratio of 1∶2 with 0.01% NaN_3_ at 37°C. Fluorescence measurements were performed using a microplate reader (FlexStation 3, DOWNINGTOWN, Pennsylvania, USA) every 30 minutes at 37.0±0.2°C. The excitation and emission wavelengths were 450 nm and 482 nm, respectively.

Aggregation kinetics was fitted using the following equation:
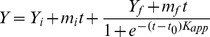
(1)


Where Y is the fluorescence intensity, *t* is time, *t*_o_ is the time to 50% of maximal fluorescence, and *k*_app_ is apparent rate constant for the growth of fibrils [41]. Kinetic data obtained from spectroscopic measurements were fitted using the nonlinear curve fitting software Original 8.0 (OriginLab, Northampton, MA, USA). In the initial fitting stage, the Simplex method was used to calculate the initial input parameters to establish the parameter region. These parameters were then used as constraints for further nonlinear curve fitting. A 0.95 confidence level target was set to constrain the quality of the curve fitting. The final fitting parameters were obtained when the value of χ*^2^* was less than 0.05, and the parameters and errors for the parameters reached a convergent and steady state.

### Western Blot Analysis of Aβ Oligomers

Each Aβ peptide was dissolved in phosphate buffer, pH 7.0, to a final concentration of 60 µM, and incubated for 0, 24, 48 and 72 hours at 37°C. Then the samples were separated by 4∼20% gradient Tricine-SDS-PAGE and transferred onto a polyvinylidene difluoride (PVDF; PE, 0.22 µm) membrane for 2 hours. The PVDF membrane was blocked using 5% nonfat milk in phosphate-buffered saline (PBS) for 1 hour and probed with primary anti-mouse monoclonal antibody (6E10, Abcam, Cambridge, UK; 1∶2000 dilution) overnight at 4°C. After probing with primary antibody, the PVDF membrane was washed three times with PBST and probed with anti-mouse secondary antibody (Sigma, Poole, UK; 1∶6000 dilution). The labeled Aβ peptides were detected using the western lighting chemiluminescence kit (GE, Pittsburgh, USA).

### Fourier-transform Infrared Spectroscopy (FT-IR)

A FT-IR spectrometer (Jasco, FT-IR/4100) equipped with an accessory was used to study the conformation of Aβ_40_ during the aggregative process. One hundred µl of 200 µM Aβ peptide solution was coated on a ZnSe crystal and kept dry overnight in a desiccator at room temperature. The spectra were recorded at a wavelength range of 1500–1800 cm^−1^ with 1 cm^−1^ intervals. The peak was identified from the first derivation of the IR spectrum in the amide I region, and the secondary structure was analyzed using Original 8.0 software.

### Transmission Electron Microscope (TEM) Analysis

A TEM (JEM-2000 EXII, JEOL, Japan) with an accelerating voltage of 100 KeV was used to analyze the morphology of Aβ peptides incubated at different time periods. Ten microliters of the Aβ peptide samples used for the aggregation assay was placed onto a carbon-coated 200 mesh copper grid (Pelco, Ca, USA). Excess solution was wicked dry with tissue paper, and the sample was negatively stained with 5 µl of 2% uranyl acetate for 30 seconds. Excess solution was wicked dry, and the grid was allowed to air dry for further TEM analysis.

### Cell Viability Assay

Human neuroblastoma SH-SY5Y cells [42] were cultured in DMEM/F12 (1∶1) supplemented (Biochrom, Berlin, Germany) with 2 mM glutamine and 10% (v/v) heat-inactivated fetal bovine serum (Biowest, Nuaillé, France) at 37°C in a humidified atmosphere containing 5% CO_2_. Cell viability was measured using 3-(4,5-dimethylthiazol-2-yl)-2,5-diphenyl tetrazolium bromide (MTT) (Sigma, Missouri, USA). All Aβ peptides were prepared as a 1 mM stock solution in trifluoroethanol. Freshly prepared stock solution was then dried under N_2_ gas and resuspended in PBS buffer, pH 7.0, to a final peptide concentration of 500 µM. The resulting solution was incubated at 25°C for 24 hours to obtain the Aβ oligomers. This peptide solution was further diluted to 30 µM for the viability assay.

In a 96-well microtiter plate, 1×10^4^ cells were placed in each well and incubated in the absence or presence of Aβ peptides in a total volume of 100 µl for 48 hours and 72 hours at 37°C in a humidified atmosphere containing 5% CO_2_ before the viability assay. Ten microliters of MTT solution was added to each well and further incubated for another 4 hours at 37°C [12]. The absorbance was measured at a wavelength of 570 nm using a microplate reader (FlexStation 3).

### Measurement of Intracellular ROS

ROS were determined using a 2′,7′-dichlorofluorescein diacetate (DCFH-DA)(Sigma, St. Louis, USA) assay in a flow cytometer (Beckman Coulter, Brea, CA, USA)**.** Cells (1×10^5^) were incubated with 30 µM of Aβ peptides for 48 hours. Before conducting flow cytometry, the cells were treated with 10 µM of DCFH-DA for 30 minutes at 37°C, followed by washing with PBS [12], [13]. Data analyses were performed using program Kaluza software, version 1.2.

### Statistical Analysis

Results were analyzed by the Student’s t-Test using Original 8.0 software. Data are expressed as mean ± standard deviation. A *p* value ≤0.05 was considered statistically significant.

## Results

### Structural Stability of Aβ_40_ and Aβ_40_ Mutations

**Figure 1:**
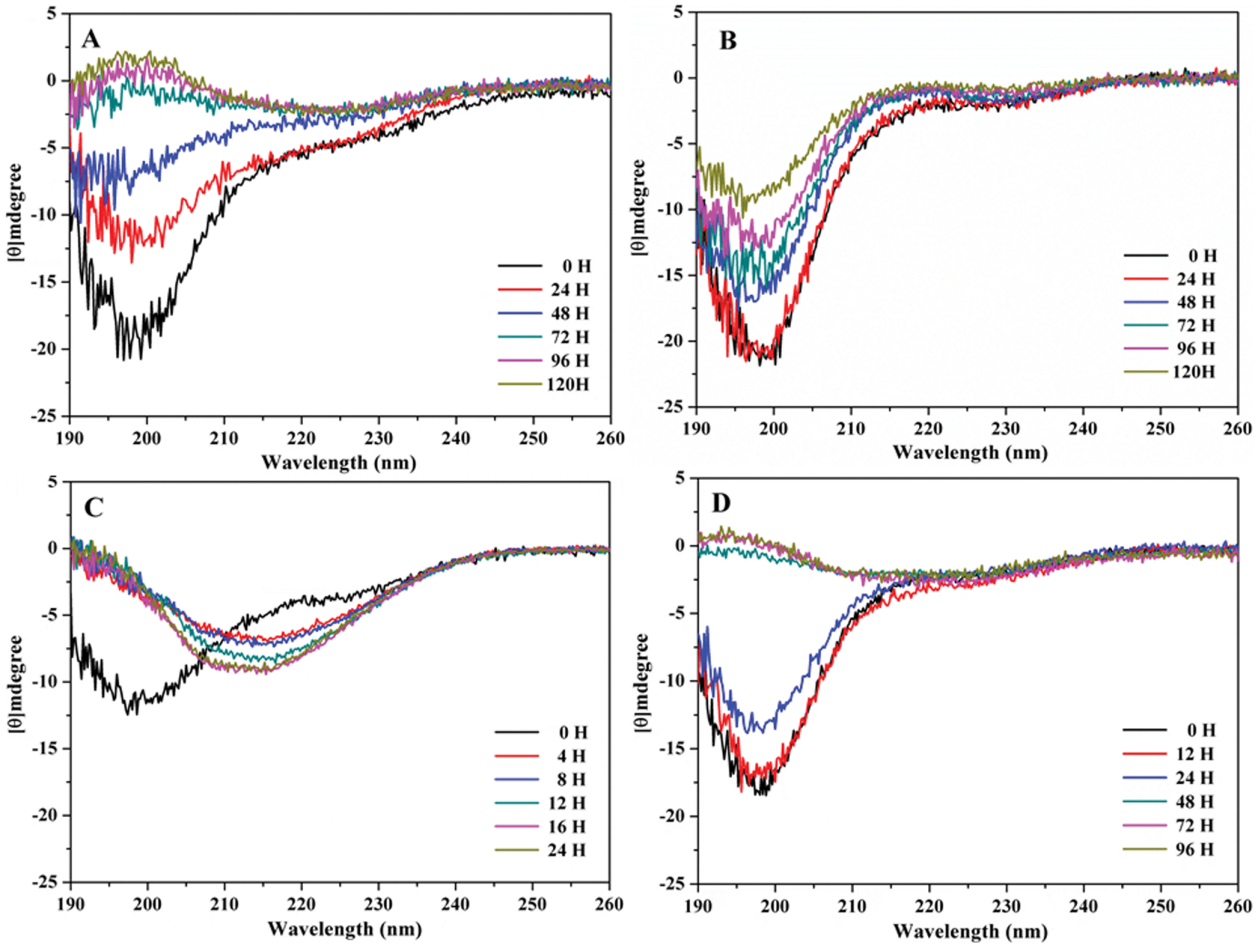
Conformational changes of Aβ_40_ using far ultraviolet circular dichroism spectra. (A) Aβ_40_, (B) Aβ_40_(L17A/F19A), (C) Aβ_40_ (E22G), (D) Aβ_40_(L17A/F19A/E22G) peptides at various incubated times. The concentration of Aβ peptides was 60 µM at pH 7.0 and 37°C.

**Figure 2:**
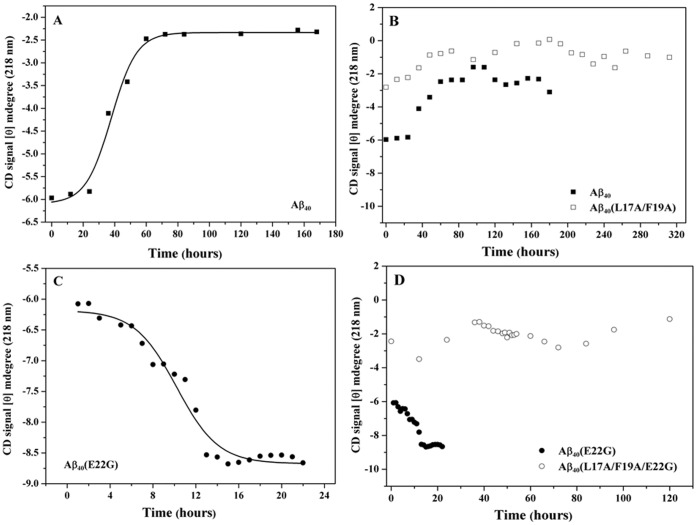
Plot of molar ellipticity at 218 nm versus incubation time. (A) Aβ_40_ (▪), (B) compare Aβ_40_ (▪) with Aβ_40_(L17A/F19A) (□), (C) Aβ_40_(E22G) (•), (D) compare Aβ_40_(E22G) with Aβ_40_(L17A/F19A/E22G) (○).

The aggregation of Aβ is accompanied by a conformational conversion from either helix or random coil to β-sheet. Therefore, the conformational state of Aβ plays a critical role in its aggregation ability and toxicity. We first applied CD spectroscopy to investigate the structural state of wild-type and Arctic-type Aβ_40_. Figure 1 (A) and (C) shows the CD spectra of wild-type Aβ_40_ and Arctic-type Aβ_40_(E22G). From the CD spectra, it can be seen that both Aβ_40_ and Arctic-type Aβ_40_(E22G) peptides were converted from random coil to β-sheet after 48 hours. The β-sheet conformation first appeared at 4 hours for Aβ_40_(E22G), while for Aβ_40_, the β-sheet structure was first observed later at 48 hours. Further analysis of β-sheet propensity (CD signal at 218 nm versus time) is shown in Figure 2 (A) and (C) for Aβ_40_ and Aβ_40_(E22G). Intensity at 218 nm increased with increased incubation time for Aβ_40_ and Aβ_40_(E22G), and the conversion rate of Aβ_40_(E22G) was faster than Aβ_40_.

### Effect of L17A and F19A on the Structural Stability of Aβ_40_ and its Mutations

Previously we replaced residues L17 and F19 with alanine, resulting in an increase in structural stability and reduction of toxicity [37]. We used the same strategy [37] to test the effect of L17A and F19A on structural stability and toxicity of the most toxic FAD mutant, Arctic-type Aβ peptide [Aβ_40_(E22G)]. Figures 1 (B) and (D) show the CD spectra for Aβ_40_(L17A/F19A) and Aβ_40_(L17A/F19A/E22G). Only Aβ_40_(L17A/F19A/E22G) was converted from random coil to β-sheet [Figure 1 (D)] after 72 hours, while Aβ_40_(L17A/F19A) remained largely in a random coiled state over a 120-hour period [Figure 1 (B)].

A plot of β-sheet propensity (CD signal at 218 nm vs. time) for Aβ_40_(L17A/F19A) and Aβ_40_(L17A/F19A/E22G) is shown in Figure 2 (B) and (D). Compared to the β-sheet propensity of Aβ_40_ and Aβ_40_(E22G), the β-sheet propensity for Aβ_40_(L17A/F19A) and Aβ_40_(L17A/F19A/E22G) was lower. The β-sheet conversion rate of Aβ_40_(L17A/F19A) and Aβ_40_(L17A/F19A/E22G) was slower than those of Aβ_40_ and Aβ_40_(E22G), suggesting that replacement of L17 and F19 with alanine increased the conformational stability of Aβ, even for Arctic-type Aβ40(E22G). Taken together, the results demonstrated that Aβ40(E22G) underwent the fastest transition. The rate of conformation conversion was the order, Aβ40 (E22G)>Aβ40> Aβ40 (L17A/F19A/E22G)>>Aβ40 (L17A/F19A).

### Aggregation Kinetics of the Aβ Peptide

**Figure 3:**
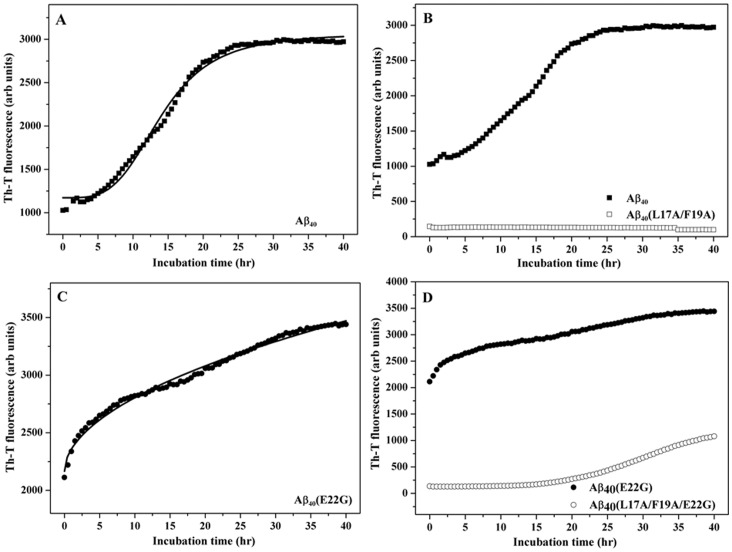
Kinetics of the aggregation process. (A) Curve fitting with Aβ_40_ (▪), (B) Group Aβ_40_ (▪) versus Aβ_40_(L17A/F19A)(□), (C) Curve fitting with Aβ_40_(E22G) (•), (D) Group Aβ_40_(E22G) (•) versus Aβ_40_(L17A/F19A/E22G) (○). The aggregation assay was performed with 60 µM Aβ peptides (Aβ: Th-T = 2∶1) at 37°C.

We further investigated the aggregation kinetics for Aβ_40_, Aβ_40_(E22G), Aβ_40_(L17A/F19A), and Aβ_40_(L17A/F19A/E22G). Figure 3 (A), (B), (C), and (D) show the aggregation process for wild-type Aβ40, Aβ40(L17A/F19A), Arctic-type Aβ40(E22G), and Aβ40(L17A/F19A/E22G) using the Th-T binding assay. Solid lines show the best fit curves using equation (1). The aggregation process of Aβ40(L17A/F19A) as shown in Figure 3 (B) contained a lag phase through the whole incubation period, suggesting that the aggregation ability of Aβ40(L17A/F19A) was reduced compared to that of wild-type Aβ40. Furthermore, the Th-T intensity of Aβ40(E22G) at the origin point was higher than that of others [Figure 3 (C)], and the aggregation profile of Arctic-type Aβ40(E22G) was most likely a hyperbolic curve instead of the typical sigmoidal curve.

Under the same conditions, the aggregation profiles of Aβ40 and Aβ_40_(L17A/F19A/E22G) [Figure 3 (B) and (D)] were the typical sigmoidal shape, although the aggregation profile of Aβ40 lacked the nucleation stage. The results show that both Aβ40 and Aβ40(L17A/F19A/E22G) underwent typical nucleation-dependent aggregation processes. This demonstrates that, similar to Aβ40(L17A/F19A), alanine replacement of L17 and F19 can reduce the aggregation rate of Arctic-type Aβ40(E22G). In Table 1, the aggregation rate of Aβ40(E22G) is 200-fold higher than that of Aβ40 and Aβ_40_(L17A/F19A/E22G), indicating that the aggregation of Arctic-type Aβ40(E22G) was faster than other Aβ_40_ peptides and may have undergone a nucleation-dependent polymerization.

**Table 1:** Calculated aggregation rates for different Aβ peptides.

Peptide	*K*_app_	*R^2^*	χ*^2^*
Aβ_40_	14.12±0.21	0.994	0.330
Aβ_40_ (E22G)	3504.20±16.43	0.998	0.009
Aβ_40_(L17A/F19A/E22G)	33.96±0.28	0.995	0.062

### Molecular State of Aβ Peptides

**Figure 4:**
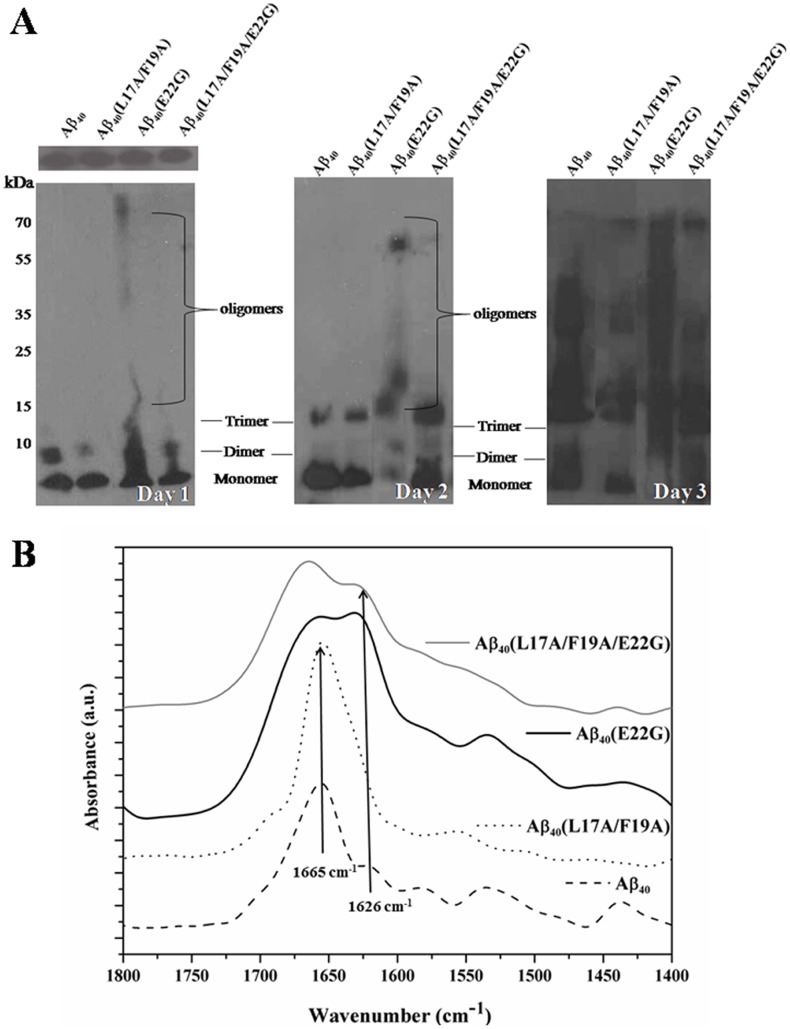
Oligomerization and fibrillization of Aβ peptides. (A) Representative western blots showing oligomeric and fibrillar Aβ peptides. Incubated Aβ peptides at 60 µM concentration at 37°C on day 0 (upper) and day 1, day 2 and day 3 Approximate molecular weights (in kD) determined using molecular weight markers are shown on the left-hand side. (B) FT-IR spectra of 0.2 mM Aβ peptides on day 3.

We further examined the molecular state of Aβs using western blot analysis. Figure 4 (A) shows the blot of Arctic-type Aβ40(E22G) and other Aβ_40_ peptides at day 0, day 1, day 2, and day 3. Consistent with the aggregation profiles, Aβ40(E22G) aggregated into more severe polymorphologies than other Aβ_40_ peptides. Compared with Aβ_40_, Aβ_40_(L17A/F19A), and Aβ_40_(L17A/F19A/E22G), Aβ40(E22G) showed a smeared band at day 1 in the western blot. This phenomenon even becomes obvious at day 3 for Aβ40(E22G). For Aβ_40_(L17A/F19A), the aggregation profile was less obvious, even at day 2 when western blotting showed slight smearing. The aggregation profiles for Aβ_40_ and Aβ_40_(L17A/F19A/E22G) were approximately similar.

The structural state of these Aβ_40_ peptides at day 3 was further confirmed using FT-IR spectra. The FT-IR spectrum area from 1400–1800 cm^−1^ were curve fitted to determine the status of possible β-sheet, random coil, and α-helix structures [43]. The spectrum area of β-sheet/aggregated is at 1610–1640 cm^−1^ and α-helix/unordered is at 1660–1685 cm^−1^ [14]. As shown in Figure 4 (B), the 1665 cm^−1^ peak of Aβ40(E22G) showed a significant shift to a 1626 cm^−1^ peak, while for Aβ_40_(L17A/F19A), the 1665 cm^−1^ peak of Aβ40(E22G) did not shift to this wavelength. The shift of the 1665 cm^−1^ peak to 1626 cm^−1^ for Aβ_40_ and Aβ_40_(L17A/F19A/E22G) was less obvious than that of Aβ40(E22G). Taken together, the results confirmed that Aβ40(E22G) aggregates more than other Aβ_40_ peptides. These results also show that L17 and F19 mutants can reduce the rate of aggregation.

### TEM Morphology of Aβ Fibrils

**Figure 5:**
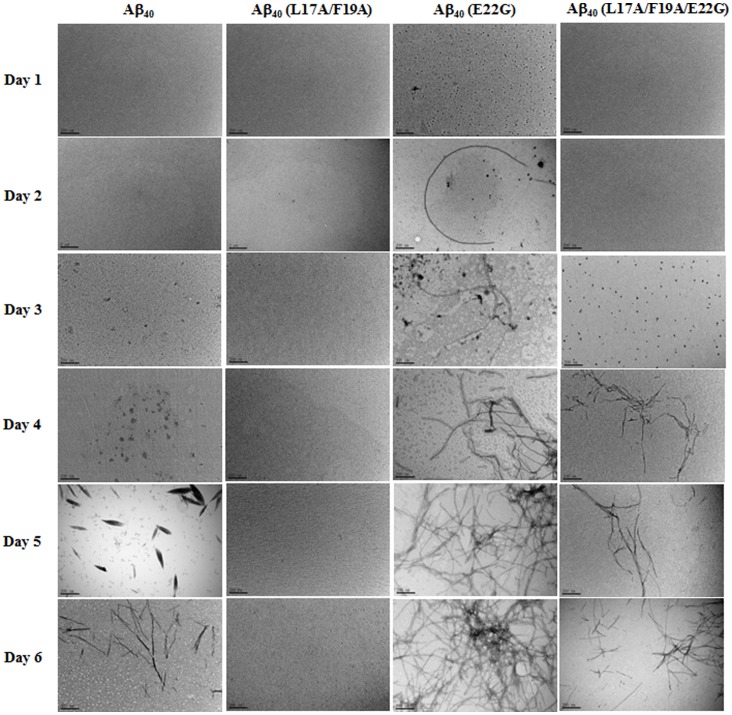
Negative stained TEM images of fibrils formed by Aβs. Fibrils formed from 60 µM peptides in phosphate buffer, pH 7.0, 37°C on day 5. All TEM images are 200,000× magnification. The scale bar indicates 200 nm.

Figure 5 shows the results of TEM for Aβ40, Aβ40(L17A/F19A), Aβ40(E22G), and Aβ40(L17A/F19A/E22G) from day 1 to day 6. TEM morphologies of Aβ40, Aβ40(E22G), and Aβ40(L17A/F19A/E22G) were all aggregated into fibrils. In contrast, no fibrils were observed for Aβ40(L17A/F19A), even at day 6. The Arctic-type Aβ40(E22G) was the fastest to form fibrils at day 2, Aβ40(L17A/F19A/E22G) formed fibrils at day 4, while wild-type Aβ40 did not form obvious fibrils until day 5. This is consistent with aggregation characterized by the other assays. The morphologic study clearly demonstrated that replacement of L17 and F19 with alanine reduced the aggregation ability of wild-type Aβ40 and Arctic-type Aβ40(E22G).

### Cell Viability

**Figure 6:**
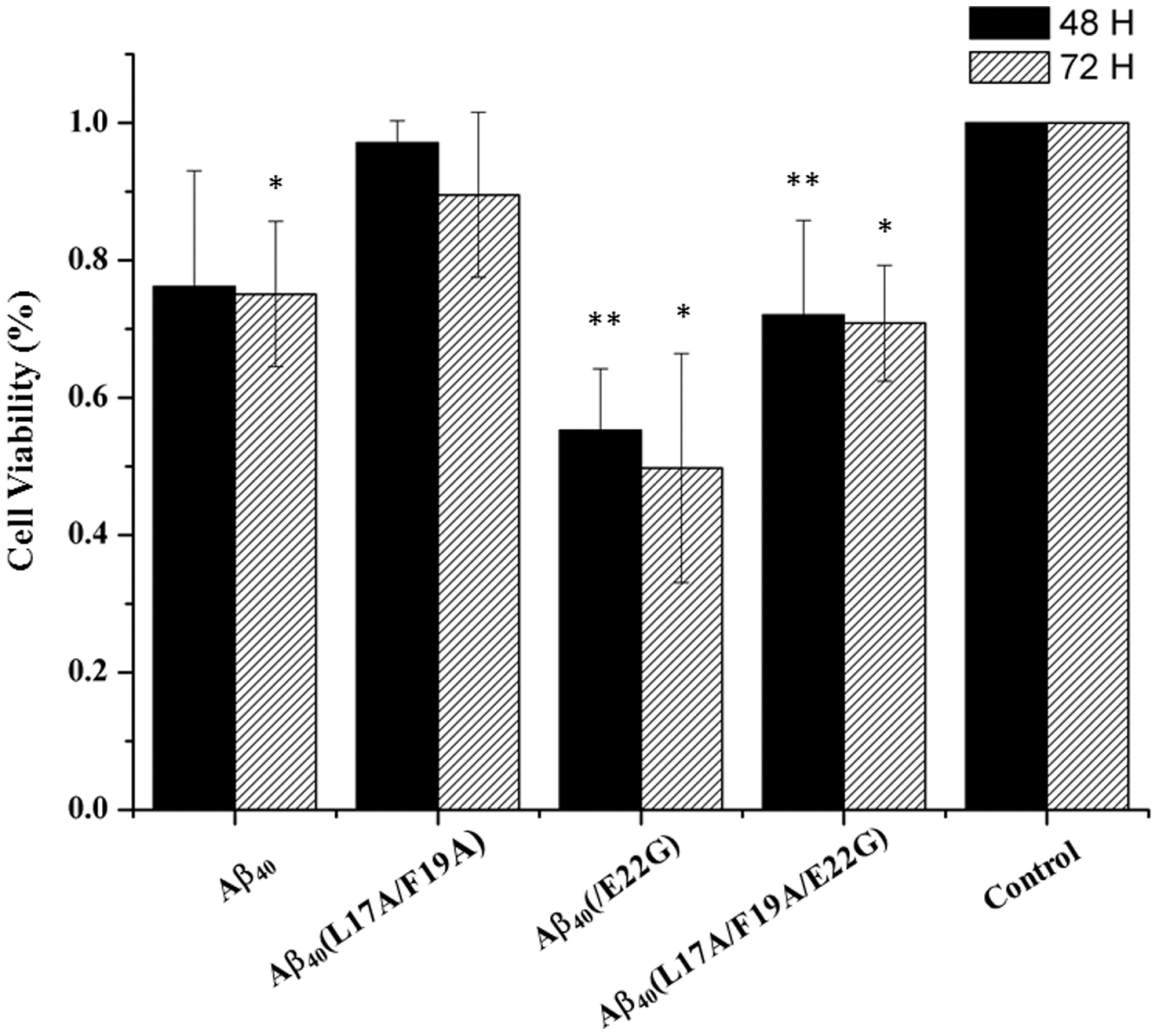
Cell viability determined by the MTT assay. Survival percentages of SHSY5Y cells incubated with 30 µM Aβs for 72 hours. Wells containing SHSY5Y cells without Aβ peptides were used as a control group.*p≤0.05 versus control was considered statistically significant. **p≤0.01.

As shown previously in this report, replacement of L17 and F19 with alanine reduced the aggregation ability and stabilized the conformation for not only wild-type Aβ40, but also for Arctic-type Aβ40(E22G). Because the conformational state and aggregation ability are linked with toxicity, the effects of L17 and F19 on toxicity induced by Arctic-type Aβ40(E22G) was further investigated. Comparative cell viability of Aβ40 peptides, including wild-type Aβ40, Aβ40(L17A/F19A), Arctic-type Aβ40(E22G), and Aβ40(L17A/F19A/E22G), was studied as shown in Figure 6. The results show the comparative cell viabilities after treatment with 30 µM of Aβ40, Aβ40(L17A/F19A), Arctic-type Aβ40(E22G), and Aβ40(L17A/F19A/E22G) peptides at 48 hours and 72 hours. Both at 48 hours and 72 hours, the toxicity induced by Aβ40(E22G) was more severe than with other peptides. Similar to a previous study [37], Aβ40(L17A/F19A) showed less toxicity than other peptides. However, toxicity induced by Aβ40(L17A/F19A/E22G) was approximately the same as Aβ40, suggesting that alanine replacement of L17 and F19 reduced the cytotoxicity induced by Aβ40(E22G), which is consistent with the aggregation rate and structural stability. Cell viability at 48 hours and 72 hours was on the order of Aβ40(L17A/F19A) >Aβ40(L17A/F19A/E22G) = Aβ40>Aβ40(E22G).

### Effect of Aβ on ROS

**Figure 7:**
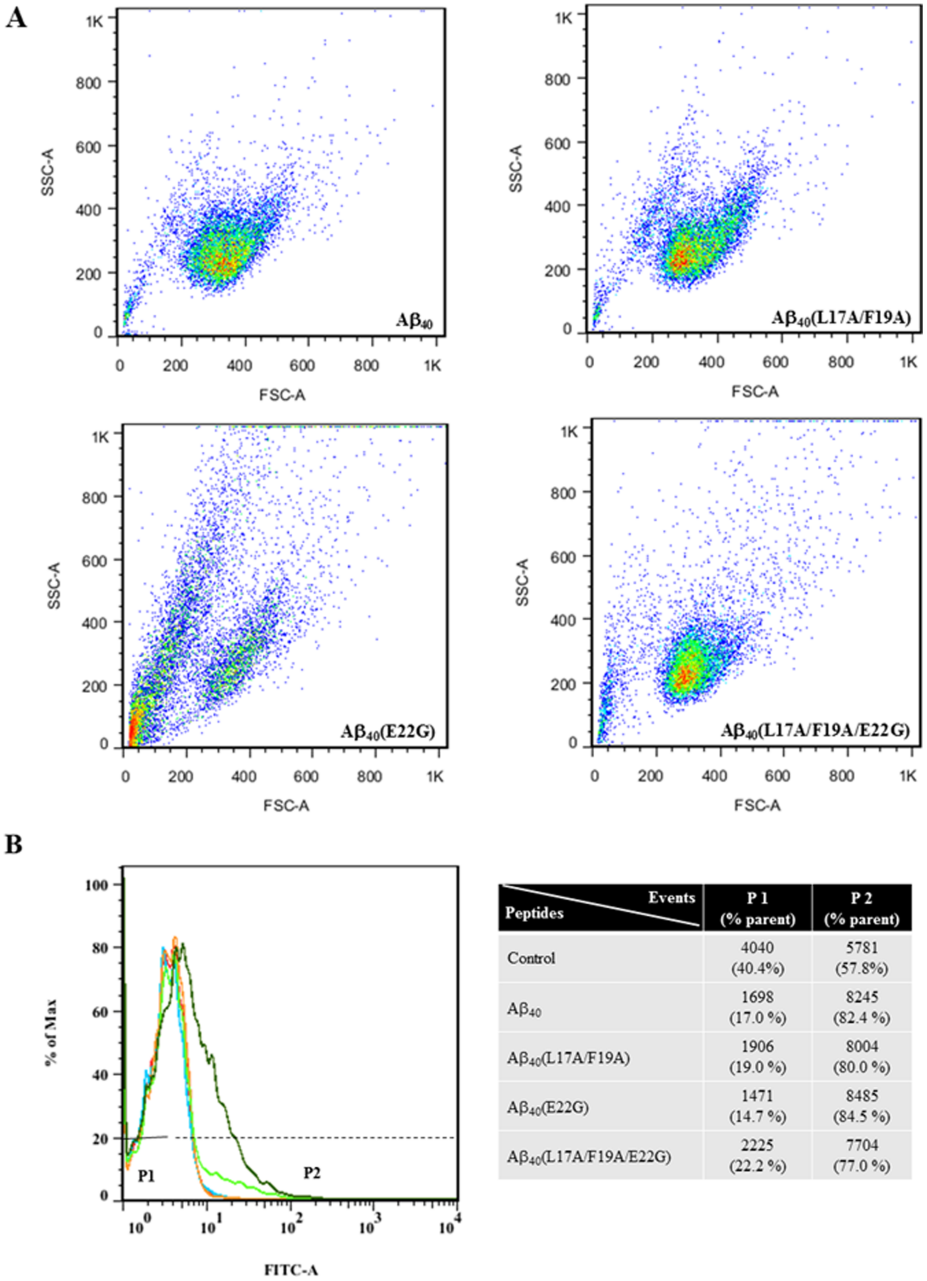
Formation of reactive oxygen species (ROS) by SHSY5Y cells. (A) Flow cytometry after DCFH-DA staining to measure ROS production of cells treated with 30 µM Aβ peptides for 48 hours. (B) Total cells are 10000 events. P1 and P2 populations are compared with control cells without peptide (red). The histogram showing Aβ_40_ is blue, Aβ_40_(L17A/F19A) is orange, Aβ_40_(E22G) is green, and Aβ_40_(L17A/F19A/E22G) is olive.

Excessive ROS generation from dysfunctional or damaged mitochondria may trigger autophagy which removes the damaged mitochondria [44]. Flow cytometry shows that the rate of cell death was greatest for Arctic-type Aβ40(E22G) [Figure 7(A)], which was consistent with the cell viability assay. Flow cytometry also showed that treatment with Aβ40(L17A/F19A/E22G) resulted in decreased ROS production (Figure 7(B), and that treatment with Arctic-type Aβ40(E22G) resulted in higher ROS that caused cell death faster than Aβ40(L17A/F19A/E22G). The same results were obtained for wild-type and Aβ40(L17A/F19A) [Figure 7(B)]. Together, the results indicated that L17A/F19A mutants could reduce ROS-induced oxidative DNA damage which could cause cell death.

## Discussion

Aggregation and toxicity of Aβ is highly correlated with its conformational states. Conformational change is a key step in the Aβ-aggregation cascade, during which the conformation of Aβ undergoes a structural conversion from either α-helix or random coil to β-sheet [15], [16]. Numerous studies have shown that prevention of conformational change can reduce Aβ aggregation. Furthermore, there have been many studies to elucidate the contribution of individual amino acid residues to helix stabilization [18], [30]. The effects of amino acid sequence variations on the conformational change of Aβ may provide useful information for further understanding the molecular mechanism of Aβ aggregation.

Previous study has shown that L18A/F19A/F20A mutant can stabilize α-helix conformation [30], [31], [33], [34]. Interestingly, our previous study identified that the amino acids L17 and F19 play a key role in the stabilization of the Aβ40 conformation, leading to the reduction of its aggregation and cytotoxicity [37]. Therefore, we would further test the effects of L17 and F19 on the conformation, aggregation, and toxicity of other Aβ40 mutants. We used the same methodologies as the previous study, constructing a rational mutation Aβ40(L17A/F19A/E22G) of Arctic-type Aβ40(E22G), and studied its conformation, aggregation ability, and cytotoxicity.

Although, unlike Aβ40(L17A/F19A), where alanine replacement of L17 and F19 of wild-type Aβ40 could completely inhibit the aggregation and toxicity, the rate of conformation conversion, aggregation ability, and toxicity of Aβ40(L17A/F19A/E22G) were greatly reduced in comparison with those of Arctic-type Aβ40(E22G), which is known to be the most toxic FAD species. Interestingly, regarding the rate of conformation conversion, aggregation ability, and induced neurotoxicity, the related properties of Aβ40(L17A/F19A/E22G) were approximately the same as wild-type Aβ40. Previously, several studies have shown that Aβ aggregation can be blocked by helix inducing reagents, such as trifluoroethanol and sodium dodecyl sulfate [45]. Moreover, it has been shown that residues 16–23 have been predicted as the region of discordant helix [30]–[34], and any factors which stabilize the conformation of this discordant helix region could prevent the aggregation of Aβ40. Our previous study identified 17–25 as the discordant helix region [36]. In another study we reported that instead of V18, F19, and F20, only L17 and F19 may be enough to stabilize the conformation of Aβ40 and inhibit its aggregation, showing that residues L17/F19 are important residues that can significantly increase the helical potential [37]. In the present study, we further demonstrated that residues L17 and F19 play crucial roles in the stabilization of Aβ40 structure. This is consistent with the hypothesis of discordant helix that residues 17–20 constitute the most sensible region for environmental changes, playing an important role in the conformational stabilization of not only Aβ40, but also other mutants such as Aβ40(E22G).

Beta-amyloid can cause cell death through the well-known process of oxidative stress. This process is an imbalance between the systemic manifestations of RNS and ROS [12], [13]. Many neurodegenerative diseases have high levels of oxidative stress, especially those affected by ROS. Our present results showed that treatment with Arctic-type Aβ40(E22G) results in more ROS than Aβ40(L17A/F19A/E22G). We also showed that treatment with Aβ40(L17A/F19A/E22G) can reduce ROS production to a level approximately equal to that produced by Aβ40. These results again demonstrate that L17 and F19 are important residues in the reduction of cytotoxicity.

The amyloidogenic differences between Aβ40(L17A/F19A) and Aβ40(L17A/F19A/E22G) further indicate that the propensity of the discordant helix is highly dependent on the sequence, because the inhibition ability of L17A/F19A on aggregation and toxicity of Aβ40(L17A/F19A/E22G) was weaker than that of Aβ40(L17A/F19A). This is obviously affected by the E22G mutation. Mutation of E22 with G22 may increase the propensity of the discordant helix of Arctic-type Aβ40(E22G) in a stronger manner than that of wild-type Aβ40. This provides a possible explanation why Arctic-type Aβ40(E22G) is more toxic than wild-type Arctic-type Aβ40, because replacement of glutamate with glycine may increase the hydrophobicity. Therefore the helix propensity is less stable for the discordant helix region of Aβ40(E22G), and as a consequence, Arctic-type Aβ40(E22G) more easily aggregates and is more toxic than wild-type Aβ40. In conclusion, our results confirm that the discordant helix region of Aβ is located at residues 17–25, which is sensitive to environmental changes. Furthermore, our study further demonstrates that residues L17 and F19 of this discordant helix may play a crucial role in the stabilization of Aβ conformation. Changes such as alanine replacement of L17 and F19 may stabilize the conformation, to diminish aggregation and reduce the neurotoxicity of Aβ40 and even Aβ40(E22G). Thus, our results provide important information about structural parameters involved in the Aβ-aggregation cascade.

## References

[1] Selkoe DJ (1991) Amyloid protein and Alzheimer’s disease. Scientific American 265: 68–71, 74–66, 78. 10.1038/scientificamerican1191-68 1785042

[2] Holtzman DM, Mobley WC (1991) Molecular studies in Alzheimer’s disease. Trends in biochemical sciences 16: 140–144. 1877089 10.1016/0968-0004(91)90056-2

[3] Standaert DG, Lee VM, Greenberg BD, Lowery DE, Trojanowski JQ (1991) Molecular features of hypothalamic plaques in Alzheimer’s disease. The American journal of pathology 139: 681–691. 1653521 PMC1886221

[4] Chui HC, Teng EL, Henderson VW, Moy AC (1985) Clinical subtypes of dementia of the Alzheimer type. Neurology 35: 1544–1550. 4058744 10.1212/wnl.35.11.1544

[5] Mucke L (2009) Neuroscience: Alzheimer’s disease. Nature 461: 895–897. 19829367 10.1038/461895a

[6] Bekris LM, Yu CE, Bird TD, Tsuang DW (2010) Genetics of Alzheimer disease. Journal of geriatric psychiatry and neurology 23: 213–227. 21045163 10.1177/0891988710383571 PMC3044597

[7] Behrouz N, Defossez A, Delacourte A, Mazzuca M (1991) The immunohistochemical evidence of amyloid diffuse deposits as a pathological hallmark in Alzheimer’s disease. Journal of gerontology 46: B209–212. 1719062 10.1093/geronj/46.6.b209

[8] Vassar R, Bennett BD, Babu-Khan S, Kahn S, Mendiaz EA, et al (1999) Beta-secretase cleavage of Alzheimer’s amyloid precursor protein by the transmembrane aspartic protease BACE. Science 286: 735–741. 10531052 10.1126/science.286.5440.735

[9] De Strooper B, Vassar R, Golde T (2010) The secretases: enzymes with therapeutic potential in Alzheimer disease. Nature reviews Neurology 6: 99–107. 20139999 10.1038/nrneurol.2009.218 PMC2879045

[10] Fawzi NL, Kohlstedt KL, Okabe Y, Head-Gordon T (2008) Protofibril assemblies of the arctic, Dutch, and Flemish mutants of the Alzheimer’s Abeta1–40 peptide. Biophysical journal 94: 2007–2016. 18032553 10.1529/biophysj.107.121467 PMC2257882

[11] Yankner BA, Lu T (2009) Amyloid beta-protein toxicity and the pathogenesis of Alzheimer disease. The Journal of biological chemistry 284: 4755–4759. 18957434 10.1074/jbc.R800018200 PMC2643502

[12] Wang H, Xu Y, Yan J, Zhao X, Sun X, et al (2009) Acteoside protects human neuroblastoma SH-SY5Y cells against beta-amyloid-induced cell injury. Brain research 1283: 139–147. 19520063 10.1016/j.brainres.2009.05.101

[13] Mei Z, Yan P, Situ B, Mou Y, Liu P (2012) Cryptotanshinione inhibits beta-amyloid aggregation and protects damage from beta-amyloid in SH-SY5Y cells. Neurochemical research 37: 622–628. 22102154 10.1007/s11064-011-0652-6

[14] Ahmed M, Davis J, Aucoin D, Sato T, Ahuja S, et al (2010) Structural conversion of neurotoxic amyloid-beta(1–42) oligomers to fibrils. Nature structural & molecular biology 17: 561–567. 10.1038/nsmb.1799 PMC2922021 20383142

[15] Kirkitadze MD, Condron MM, Teplow DB (2001) Identification and characterization of key kinetic intermediates in amyloid beta-protein fibrillogenesis. Journal of molecular biology 312: 1103–1119. 11580253 10.1006/jmbi.2001.4970

[16] Xu Y, Shen J, Luo X, Zhu W, Chen K, et al (2005) Conformational transition of amyloid beta-peptide. Proceedings of the National Academy of Sciences of the United States of America 102: 5403–5407. 15800039 10.1073/pnas.0501218102 PMC556260

[17] Bitan G, Kirkitadze MD, Lomakin A, Vollers SS, Benedek GB, et al (2003) Amyloid beta -protein (Abeta) assembly: Abeta 40 and Abeta 42 oligomerize through distinct pathways. Proceedings of the National Academy of Sciences of the United States of America 100: 330–335. 12506200 10.1073/pnas.222681699 PMC140968

[18] Liao MQ, Tzeng YJ, Chang LY, Huang HB, Lin TH, et al (2007) The correlation between neurotoxicity, aggregative ability and secondary structure studied by sequence truncated Abeta peptides. FEBS letters 581: 1161–1165. 17328898 10.1016/j.febslet.2007.02.026

[19] Liu R, McAllister C, Lyubchenko Y, Sierks MR (2004) Residues 17–20 and 30–35 of beta-amyloid play critical roles in aggregation. Journal of neuroscience research 75: 162–171. 14705137 10.1002/jnr.10859

[20] Goedert M, Spillantini MG (2006) A century of Alzheimer’s disease. Science 314: 777–781. 17082447 10.1126/science.1132814

[21] Hendriks L, van Duijn CM, Cras P, Cruts M, Van Hul W, et al (1992) Presenile dementia and cerebral haemorrhage linked to a mutation at codon 692 of the beta-amyloid precursor protein gene. Nature genetics 1: 218–221. 1303239 10.1038/ng0692-218

[22] Kamino K, Orr HT, Payami H, Wijsman EM, Alonso ME, et al (1992) Linkage and mutational analysis of familial Alzheimer disease kindreds for the APP gene region. American journal of human genetics 51: 998–1014. 1415269 PMC1682859

[23] Nilsberth C, Westlind-Danielsson A, Eckman CB, Condron MM, Axelman K, et al (2001) The ‘Arctic’ APP mutation (E693G) causes Alzheimer’s disease by enhanced Abeta protofibril formation. Nature neuroscience 4: 887–893. 11528419 10.1038/nn0901-887

[24] Natte R, Maat-Schieman ML, Haan J, Bornebroek M, Roos RA, et al (2001) Dementia in hereditary cerebral hemorrhage with amyloidosis-Dutch type is associated with cerebral amyloid angiopathy but is independent of plaques and neurofibrillary tangles. Annals of neurology 50: 765–772. 11761474 10.1002/ana.10040

[25] Szczesna-Cordary D, Guzman G, Zhao J, Hernandez O, Wei J, et al (2005) The E22K mutation of myosin RLC that causes familial hypertrophic cardiomyopathy increases calcium sensitivity of force and ATPase in transgenic mice. Journal of cell science 118: 3675–3683. 16076902 10.1242/jcs.02492

[26] Grabowski TJ, Cho HS, Vonsattel JP, Rebeck GW, Greenberg SM (2001) Novel amyloid precursor protein mutation in an Iowa family with dementia and severe cerebral amyloid angiopathy. Annals of neurology 49: 697–705. 11409420 10.1002/ana.1009

[27] Murakami K, Irie K, Morimoto A, Ohigashi H, Shindo M, et al (2002) Synthesis, aggregation, neurotoxicity, and secondary structure of various A beta 1–42 mutants of familial Alzheimer’s disease at positions 21–23. Biochemical and biophysical research communications 294: 5–10. 12054732 10.1016/S0006-291X(02)00430-8

[28] Peralvarez-Marin A, Mateos L, Zhang C, Singh S, Cedazo-Minguez A, et al (2009) Influence of residue 22 on the folding, aggregation profile, and toxicity of the Alzheimer’s amyloid beta peptide. Biophysical journal 97: 277–285. 19580765 10.1016/j.bpj.2009.04.017 PMC2711388

[29] Norlin N, Hellberg M, Filippov A, Sousa AA, Grobner G, et al (2012) Aggregation and fibril morphology of the Arctic mutation of Alzheimer’s Abeta peptide by CD, TEM, STEM and in situ AFM. Journal of structural biology 180: 174–189. 22750418 10.1016/j.jsb.2012.06.010 PMC3466396

[30] Paivio A, Nordling E, Kallberg Y, Thyberg J, Johansson J (2004) Stabilization of discordant helices in amyloid fibril-forming proteins. Protein science : a publication of the Protein Society 13: 1251–1259. 15096631 10.1110/ps.03442404 PMC2286751

[31] Kallberg Y, Gustafsson M, Persson B, Thyberg J, Johansson J (2001) Prediction of amyloid fibril-forming proteins. The Journal of biological chemistry 276: 12945–12950. 11134035 10.1074/jbc.M010402200

[32] Nerelius C, Sandegren A, Sargsyan H, Raunak R, Leijonmarck H, et al (2009) Alpha-helix targeting reduces amyloid-beta peptide toxicity. Proceedings of the National Academy of Sciences of the United States of America 106: 9191–9196. 19458258 10.1073/pnas.0810364106 PMC2695042

[33] Ito M, Johansson J, Stromberg R, Nilsson L (2011) Unfolding of the amyloid beta-peptide central helix: mechanistic insights from molecular dynamics simulations. PloS one 6: e17587. 21408230 10.1371/journal.pone.0017587 PMC3049775

[34] Ito M, Johansson J, Stromberg R, Nilsson L (2012) Effects of ligands on unfolding of the amyloid beta-peptide central helix: mechanistic insights from molecular dynamics simulations. PloS one 7: e30510. 22291970 10.1371/journal.pone.0030510 PMC3264620

[35] Hatip FF, Suenaga M, Yamada T, Matsunaga Y (2009) Reversal of temperature-induced conformational changes in the amyloid-beta peptide, Abeta40, by the beta-sheet breaker peptides 16–23 and 17–24. British journal of pharmacology 158: 1165–1172. 19785651 10.1111/j.1476-5381.2009.00384.x PMC2785537

[36] Wang CC, Huang HB, Tsay HJ, Shiao MS, Wu WJ, et al (2012) Characterization of Abeta aggregation mechanism probed by congo red. Journal of biomolecular structure & dynamics 30: 160–169. 22702727 10.1080/07391102.2012.677767

[37] Chen YR, Huang HB, Lo CJ, Wang CC, Su CL, et al (2011) Abeta40(L17A/F19A) mutant diminishes the aggregation and neurotoxicity of Abeta40. Biochemical and biophysical research communications 405: 91–95. 21216230 10.1016/j.bbrc.2010.12.133

[38] Lee EK, Hwang JH, Shin DY, Kim DI, Yoo YJ (2005) Production of recombinant amyloid-beta peptide 42 as an ubiquitin extension. Protein expression and purification 40: 183–189. 15721787 10.1016/j.pep.2004.12.014

[39] Lobley A, Whitmore L, Wallace BA (2002) DICHROWEB: an interactive website for the analysis of protein secondary structure from circular dichroism spectra. Bioinformatics 18: 211–212. 11836237 10.1093/bioinformatics/18.1.211

[40] Whitmore L, Wallace BA (2004) DICHROWEB, an online server for protein secondary structure analyses from circular dichroism spectroscopic data. Nucleic acids research 32: W668–673. 15215473 10.1093/nar/gkh371 PMC441509

[41] Nielsen L, Khurana R, Coats A, Frokjaer S, Brange J, et al (2001) Effect of environmental factors on the kinetics of insulin fibril formation: elucidation of the molecular mechanism. Biochemistry 40: 6036–6046. 11352739 10.1021/bi002555c

[42] Biedler JL, Roffler-Tarlov S, Schachner M, Freedman LS (1978) Multiple neurotransmitter synthesis by human neuroblastoma cell lines and clones. Cancer research 38: 3751–3757. 29704

[43] Tamm LK, Tatulian SA (1997) Infrared spectroscopy of proteins and peptides in lipid bilayers. Quarterly reviews of biophysics 30: 365–429. 9634652 10.1017/s0033583597003375

[44] Chen Y, McMillan-Ward E, Kong J, Israels SJ, Gibson SB (2008) Oxidative stress induces autophagic cell death independent of apoptosis in transformed and cancer cells. Cell death and differentiation 15: 171–182. 17917680 10.1038/sj.cdd.4402233

[45] Benseny-Cases N, Klementieva O, Cladera J (2012) In vitroOligomerization and Fibrillogenesis of Amyloid-beta Peptides. Sub-cellular biochemistry 65: 53–74. 23224999 10.1007/978-94-007-5416-4_3

